# Phenytoin inhibits necroptosis

**DOI:** 10.1038/s41419-018-0394-3

**Published:** 2018-03-02

**Authors:** Anne von Mässenhausen, Wulf Tonnus, Nina Himmerkus, Simon Parmentier, Danish Saleh, Diego Rodriguez, Jiraporn Ousingsawat, Rosalind L. Ang, Joel M. Weinberg, Ana B. Sanz, Alberto Ortiz, Adrian Zierleyn, Jan Ulrich Becker, Blandine Baratte, Nathalie Desban, Stéphane Bach, Ina Maria Schiessl, Shoko Nogusa, Siddharth Balachandran, Hans Joachim Anders, Adrian T. Ting, Markus Bleich, Alexei Degterev, Karl Kunzelmann, Stefan R. Bornstein, Douglas R. Green, Christian Hugo, Andreas Linkermann

**Affiliations:** 1 Division of Nephrology, Medical Clinic 3, University Hospital Carl Gustav Carus, Technical University Dresden, Dresden, Germany; 2 Department of Physiology, University of Kiel, Kiel, Germany; 3 Sackler School of Graduate Biomedical Sciences, Tufts University School of Medicine, Boston, MA USA; 4 Department of Immunology, St. Jude Medical Research Hospital, Memphis, TN USA; 5 Institute for Physiology, University of Regensburg, Regensburg, Germany; 6 Immunology Institute, Icahn School of Medicine at Mount Sinai, New York, NY 10029 USA; 7 Department of Internal Medicine, University of Michigan, Ann Arbor, MI United States; 8 Nephrology, IIS-Fundacion Jimenez Diaz UAM, FRIAT and REDINREN, Madrid, Spain; 9 Institute of Pathology, University of Cologne, Cologne, Germany; 10 Protein Phosphorylation and Human Disease Laboratory, Station Biologique de Roscoff UPMC Univ Paris 06 CNRS USR3151, CS 90074, Sorbonne Universités, 29688 Roscoff Cedex, France; 11 Blood Cell Development and Function Program, Fox Chase Cancer Center, Philadelphia, PA USA; 12 Medizinische Klinik und Poliklinik IV, Klinikum der LMU München, Munich, Germany

## Abstract

Receptor-interacting protein kinases 1 and 3 (RIPK1/3) have best been described for their role in mediating a regulated form of necrosis, referred to as necroptosis. During this process, RIPK3 phosphorylates mixed lineage kinase domain-like (MLKL) to cause plasma membrane rupture. RIPK3-deficient mice have recently been demonstrated to be protected in a series of disease models, but direct evidence for activation of necroptosis in vivo is still limited. Here, we sought to further examine the activation of necroptosis in kidney ischemia-reperfusion injury (IRI) and from TNFα-induced severe inflammatory response syndrome (SIRS), two models of RIPK3-dependent injury. In both models, MLKL-ko mice were significantly protected from injury to a degree that was slightly, but statistically significantly exceeding that of RIPK3-deficient mice. We also demonstrated, for the first time, accumulation of pMLKL in the necrotic tubules of human patients with acute kidney injury. However, our data also uncovered unexpected elevation of blood flow in MLKL-ko animals, which may be relevant to IRI and should be considered in the future. To further understand the mode of regulation of cell death by MLKL, we screened a panel of clinical plasma membrane channel blockers and we found phenytoin to inhibit necroptosis. However, we further found that phenytoin attenuated RIPK1 kinase activity in vitro, likely due to the hydantoin scaffold also present in necrostatin-1, and blocked upstream necrosome formation steps in the cells undergoing necroptosis. We further report that this clinically used anti-convulsant drug displayed protection from kidney IRI and TNFα-induces SIRS in vivo. Overall, our data reveal the relevance of RIPK3-pMLKL regulation for acute kidney injury and identifies an FDA-approved drug that may be useful for immediate clinical evaluation of inhibition of pro-death RIPK1/RIPK3 activities in human diseases.

## Introduction

The prevention of necrosis represents a major unmet clinical need^1^. Loss of function of necrotic cells and the immunogenicity of damage-associated molecular patterns drive autoimmunity, ischemic, and toxic organ damages and cancers^2^. The recent understanding of necrosis as a series of regulated cell death pathways (necroptosis^3,4^, ferroptosis^5^, pyroptosis^6,7^ and others) allows targeting of necrosis. Necroptosis is the best studied pathway of regulated necrosis and is mediated by RIPK1-mediated activation of RIPK3^8–10^. One of these target proteins, mixed lineage kinase domain like (MLKL), is required for necroptosis^11,12^. However, different RIPK3-targets have recently been demonstrated to contribute to immune modulation in an RIPK3-dependent, but MLKL-independent manner^13,14^. It is therefore unclear if necroptosis or RIPK3-activation independent of cell death modulates the immune system and explains the protective effects of RIPK3-deficient mice in ischemic injury and other diseases, such as the TNFα-mediated shock (SIRS) or ischemia-reperfusion injury (IRI).

Here, we employed MLKL-deficient mice to demonstrate that necroptosis, and not cell death independent functions of RIPK3, contribute to renal IRI and SIRS. In a small-scale screen for plasma membrane channel inhibitors, we found the anticonvulsant phenytoin to prevent necroptosis in vitro and in vivo, potentially offering a therapeutic opportunity for the interference with necroptosis. Finally, we directly detected pMLKL positivity in human biopsy samples obtained from acute kidney injury (AKI) patients and thereby support the pathophysiological evidence for necroptosis in humans.

## Results

### MLKL-deficient mice exhibit prolonged survival following TNFα-induced shock

**Fig. 1:**
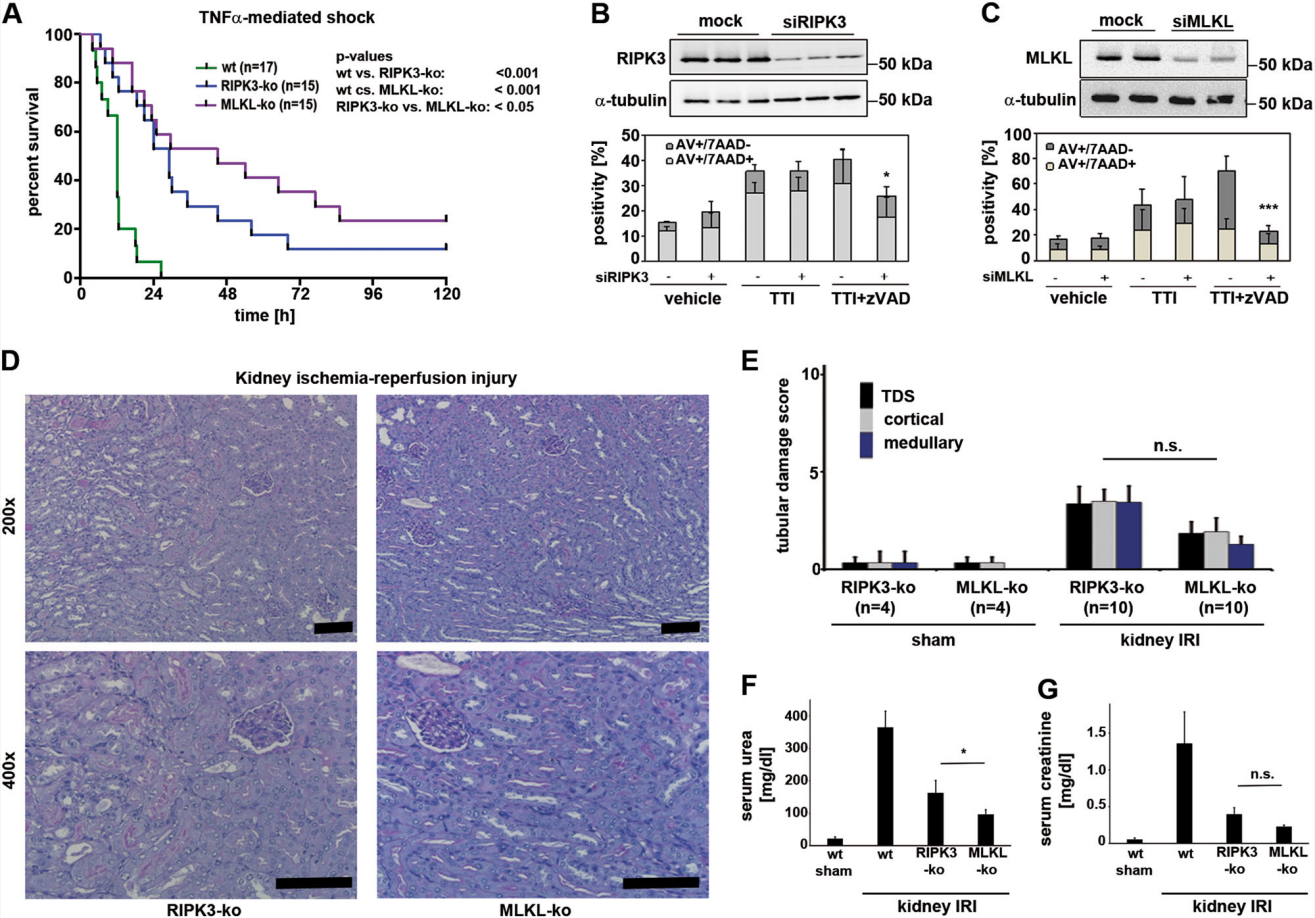
MLKL mediates septic and ischemic injury. **a** Survival after injection of recombinant human TNFα into wt, RIPK3-ko or MLKL-ko mice. **b**−**c** siRNA-mediated knockdown of RIPK3 or MLKL protects murine renal tubular cells (MCT) from TNFα/TWEAK/IFNγ(TTI) + zVAD-fmk (zVAD)-induced necroptosis 24 h after induction of cell death. Western blots for RIPK3 and MLKL indicate the efficiency of the siRNA-mediated knockdown. **d**−**g** Head-to-head comparison of RIPK3-deficient mice to MLKL-deficient mice in the model of renal ischemia-reperfusion injury (IRI). Eight-week-old RIPK3-ko and MLKL-ko mice were subjected to 30 min of renal pedicle clamping before the onset of reperfusion. Histological changes (**d**, scale bars = 50 µm) were quantified (**e**) 48 h later by employing a renal tubular damage score (TDS, see Methods for details), and functional markers of acute kidney injury (serum urea (**f**) and serum creatinine (**g**)) were measured. No statistically significant differences were detected between RIPK3-ko and MLKL-ko mice in any of these models. Statistical significance was indicated: n.s.: not statistically significant, *p* < 0.05 (*), *p* < 0.01 (**), *p* < 0.001 (***), *n* = 10−17 per group

RIPK3-deficient mice have been reported by us and others to be partially protected from SIRS induced by intravenous injection of recombinant TNFα^15,16^, but it remained unclear which downstream RIPK3 target mediates this protection. Fig. 1a demonstrates that MLKL-deficient mice phenocopy the partial protection of RIPK3-ko mice. Interestingly, MLKL-deficient mice exhibited a significantly different level of protection not only when compared with wild-type mice, but also in comparison with the RIPK3-ko animals. However, as most of these mice die, the protective effect of the MLKL-ko mice is much less protected than RIPK1-kinase dead knock-in mice^17–19^, or caspase-8/RIPK3-dko mice^20^.

### MLKL-deficient mice phenocopy the protection from ischemia-reperfusion injury observed in RIPK3-deficient mice

Human AKI is characterized by various morphologically different forms of tubular necrosis (Figure S1), and targeting necrosis may be of particular benefit for patients suffering from AKI. To study the best characterized mode of regulated necrosis, necroptosis, we knocked down RIPK3 or MLKL from murine kidney cells (MCTs) and found protection from necroptosis induced by TNFα/TWEAK/Interferon-γ in the presence of the pan-caspase inhibitor zVAD-fmk (Fig. 1b, c). In addition, in a well-established model of murine IRI, MLKL-deficient mice exhibited less structural damage (Fig. 1d, e) and less severe functional AKI (Fig. 1f, g) when compared with wild-type mice. Given the previously observed protection of RIPK3-ko in renal IRI^21^, we included RIPK3-deficient mice into this study. Similar to the results in the SIRS model (Fig. 1a), we documented a trend towards superior protection in MLKL-ko mice in histology, renal damage scores, and functional markers of AKI such as serum creatinine (Fig. 1d, e, g). However, this trend reached statistical significance in the case of serum urea concentrations (Fig. 1f). These data indicate that MLKL mediates necroptosis in SIRS and renal IRI and suggest that RIPK3-dependent, MLKL-independent effects are of minor importance in these models.

### Detection of phospho-MLKL in human biopsies obtained from patients with acute kidney injury

**Fig. 2:**
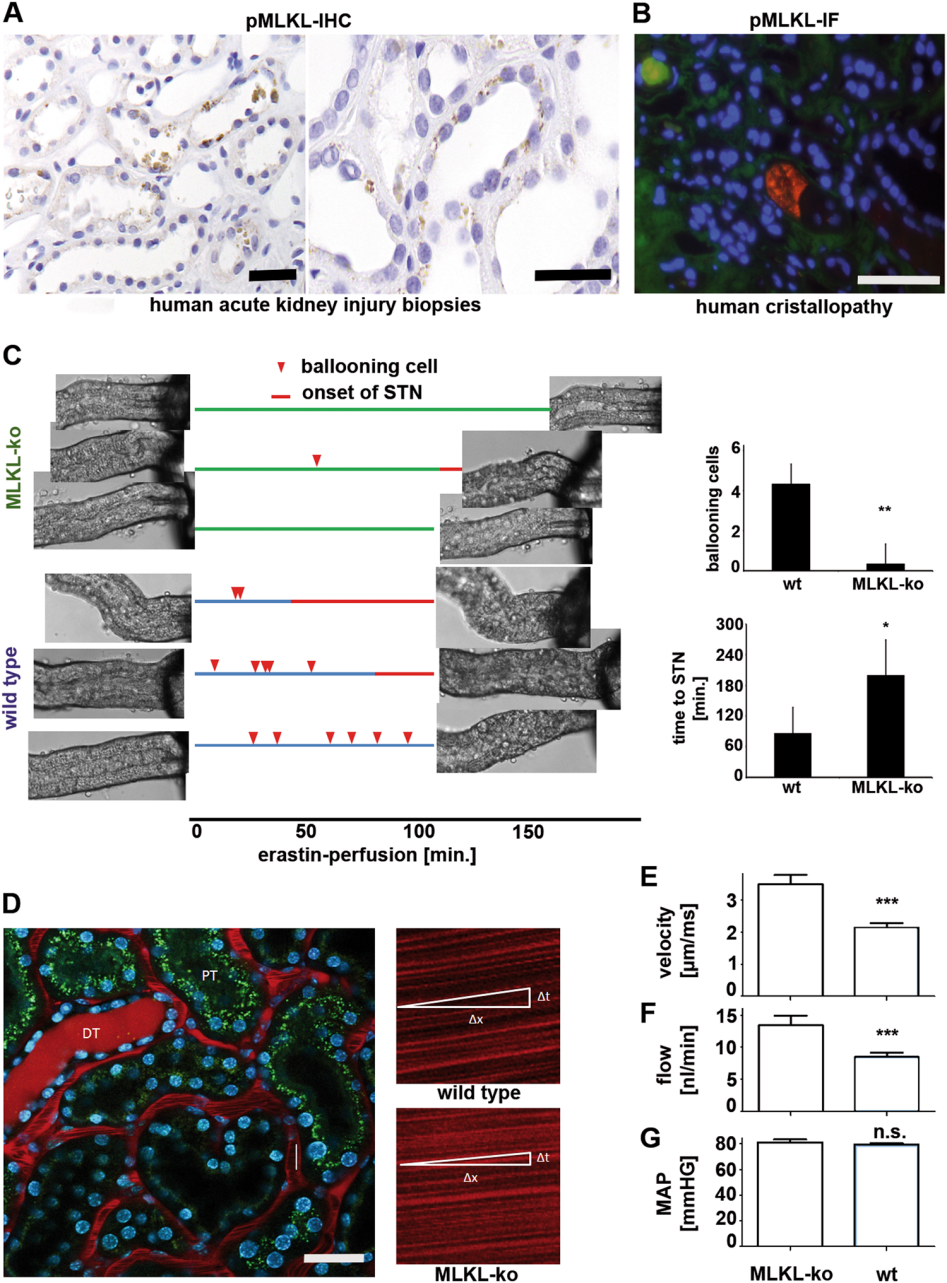
Detection of the human relevance of necroptosis and the role of MLKL. **a** Human kidney transplant biopsies obtained from patients 4 days following ischemia-reperfusion injury were stained for pMLKL. **b** Immunofluorescence of a renal biopsy sample taken from a patient with diagnosed crystallopathy. **c** Freshly isolated hand-picked kidney tubules from either wt or MLKL-ko mice were perfused with the ferroptosis-inducing compound erastin and video-monitored for morphological changes (compare Video S1). Ballooning cells are indicated as spikes on the time charts corresponding to each experiment. Change of color of the time chart indicates the onset of synchronized tubular necrosis (STN) of all cells of the tubules. Note that MLKL-ko mice exhibit highly significantly less ballooning cells and longer time to the onset of STN. **d** High-resolution intravital microscopy of MLKL-ko and wild-type littermates was employed to measure **e** velocity and **f** flow in the peritubular capillaries. In addition, mean arterial pressure (MAP) was quantified. Note the strong reduction in peritubular flow. Statistical significance was indicated: n.s.: not statistically significant, *p* < 0.05 (*), *p* < 0.01 (**), *p* < 0.001 (***). Scale bars = 50 µm

Until today, and despite the broad general interest, activity of the necroptosis pathway has been detected in humans only in kidney transplant biopsies, but expression of pMLKL in those studies was not associated with a morphological phenotype of necrosis^22^. Given the very clear contribution of necrosis to human AKI (Figure S1A-D)^23^, we investigated biopsies obtained from patients that suffered from AKI for pMLKL-positivity using a previously described method^22^. Employing a specific monoclonal antibody of a human pMLKL, we found pMLKL positivity in about 5% of AKI samples in 10−15% of the affected tubules (Fig. 2a). To our knowledge, these samples exhibit the first detection of necroptosis in human conditions as they are clearly associated with a necrotic morphology. However, several samples showed necrotic tubules the majority of which did not stain positive for pMLKL, suggesting the parallel existence of other pathways of regulated necrosis. To understand the function of pMLKL in disease models, we employed a previously established ex vivo approach in which we use hand-picked primary kidney tubules. Whereas MLKL-deficient tubules are not protected from hypoxia-reoxygenation per se (Figure S2A-C), they appear to undergo delayed synchronized tubular necrosis (STN) upon erastin-stimulation (Fig. 2c), a compound that induces another type of regulated necrosis referred to as ferroptosis^24,25^. Taken together, these experiments suggest a role for pMLKL in SIRS, renal IRI in mice and humans and erastin-induced STN in mice, but not in isolated settings of hypoxia-driven damage ex vivo. Given (i) this virtual discrepancy and (ii) our previous observations of either the necroptosis-inhibitor Nec-1 or RIPK3-deficiency to directly influence on capillary blood flow^21,26^, we hypothesized that changes in the vasculature of MLKL-deficient mice might contribute to the observed protective effects in the disease models.

### MLKL-deficient mice spontaneously exhibit accelerated peritubular blood flow

Capillary endothelial cells are of interest in both renal IRI and SIRS. We investigated MLKL-deficient mice in intravital microscopy in a strictly double-blinded fashion (Fig. 2d). Strikingly, MLKL-ko mice exhibit much higher blood velocity and stronger overall flow in the peritubular capillaries (Fig. 2e, f). Importantly in this setting, the mean arterial blood pressure was indistinguishable from either group of littermates (Fig. 2g). This phenomenon might contribute to the protection from IRI, and may also be of importance in the SIRS model. Taken together, our results suggest a combined role of MLKL in (i) directly mediating the damage to the parenchymal cells and (ii) regulating blood flow. The mechanism of the latter remains to be investigated in more detail.

### A cellular hypothesis-driven screen identifies phenytoin to prevent necroptosis

**Fig. 3:**
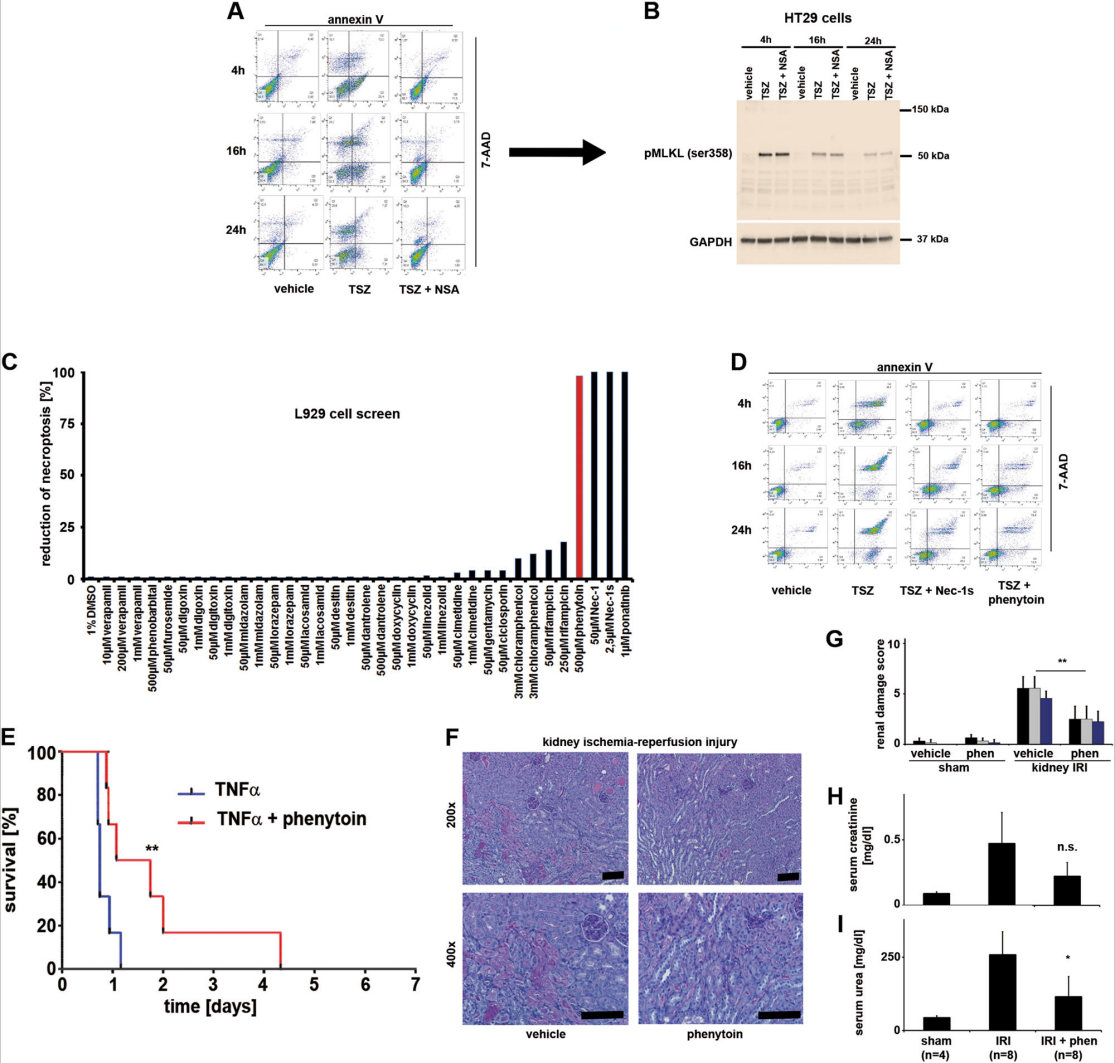
A cell-based screen identifies phenytoin as an inhibitor of necroptosis. **a**,** b** HT29 cells were treated with TNFα/smac mimetic/zVAD-fmk to induce necrosulfonamide (NSA)-sensitive necroptosis over 4, 16 and 24 h. Cell cultures were split for FACS analysis (**a**) and western blotting (**b**). Note that cells that exhibit pMLKL-positivity in western blots (16 h with NSA) did neither lose plasma membrane integrity nor became positive for annexin V between 16 and 24 h. **c** Hypothesis-based screen for clinically available inhibitors of plasma membrane channels based on 7-AAD/annexinV positivity in L929 cells 48 h following TZ-treatment. Nec-1, Nec-1s, and ponatinib served as positive controls. **d** Verification of phenytoin in HT29 cells as an inhibitor of TSZ-induced necroptosis in direct comparison with Nec-1s. **e** Phenytoin prolongs survival after TNFα-induced SIRS. **e**−**h** Phenytoin significantly protects from renal ischemia-reperfusion injury. Statistical significance was indicated: n.s.: not statistically significant, *p* < 0.05 (*), *p* < 0.01 (**), *n* = 8 mice per group unless otherwise indicated. Scale bars = 50 µm

Based on the above-mentioned findings and the recent observation that necroptosis regulation downstream of pMLKL by the ESCRT-III complex critically modulates the kinetics of necroptotic cell death^22^, we challenged the current model in which a pMLKL inevitably results in necroptosis. To test this, we treated HT29 cells with TSZ to induce necroptosis and added the MLKL-inhibitor necrosulfonamide (NSA)^11^. We investigated three time points up to 24 h and split the cell culture to be read out by fluorescence-activated cell sorting (FACS) and western blot. As demonstrated in Fig. 3a, b, within 4 h of TSZ-treatment, NSA-incubated cells clearly became positive for pMLKL, but all of these cells were negative for both annexin V and 7AAD. In the progression of this experiment until 24 h, pMLKL-positive cells did not undergo cell death during this time despite a strong pMLKL signal, clearly demonstrating that positivity for pMLKL is not sufficient to drive necroptosis.

If pMLKL is required but not sufficient for necroptosis execution and the swelling of the cells, we tested the hypothesis that plasma membrane channels may be involved downstream of pMLKL by a small-scale, hypothesis-driven screen with FDA-approved drugs that are known to exert inhibitory effects on plasma membrane channels (Fig. 3c). We found the anticonvulsive drug phenytoin (Dilantin) to protect from necroptosis as efficiently as Nec-1 24 h after necroptosis induction (Fig. 3c, d) and confirmed this result in various necroptosis-sensitive cell lines (Figure S3). We next investigated phenytoin in the in vivo models. In the SIRS model, phenytoin slightly, but significantly delayed the time to death after TNF-injection (Fig. 3f), and in the renal IRI model, application of phenytoin attenuated histological damage (Fig. 3g, h) and functional markers of AKI (Fig. 3i, j).

**Fig. 4:**
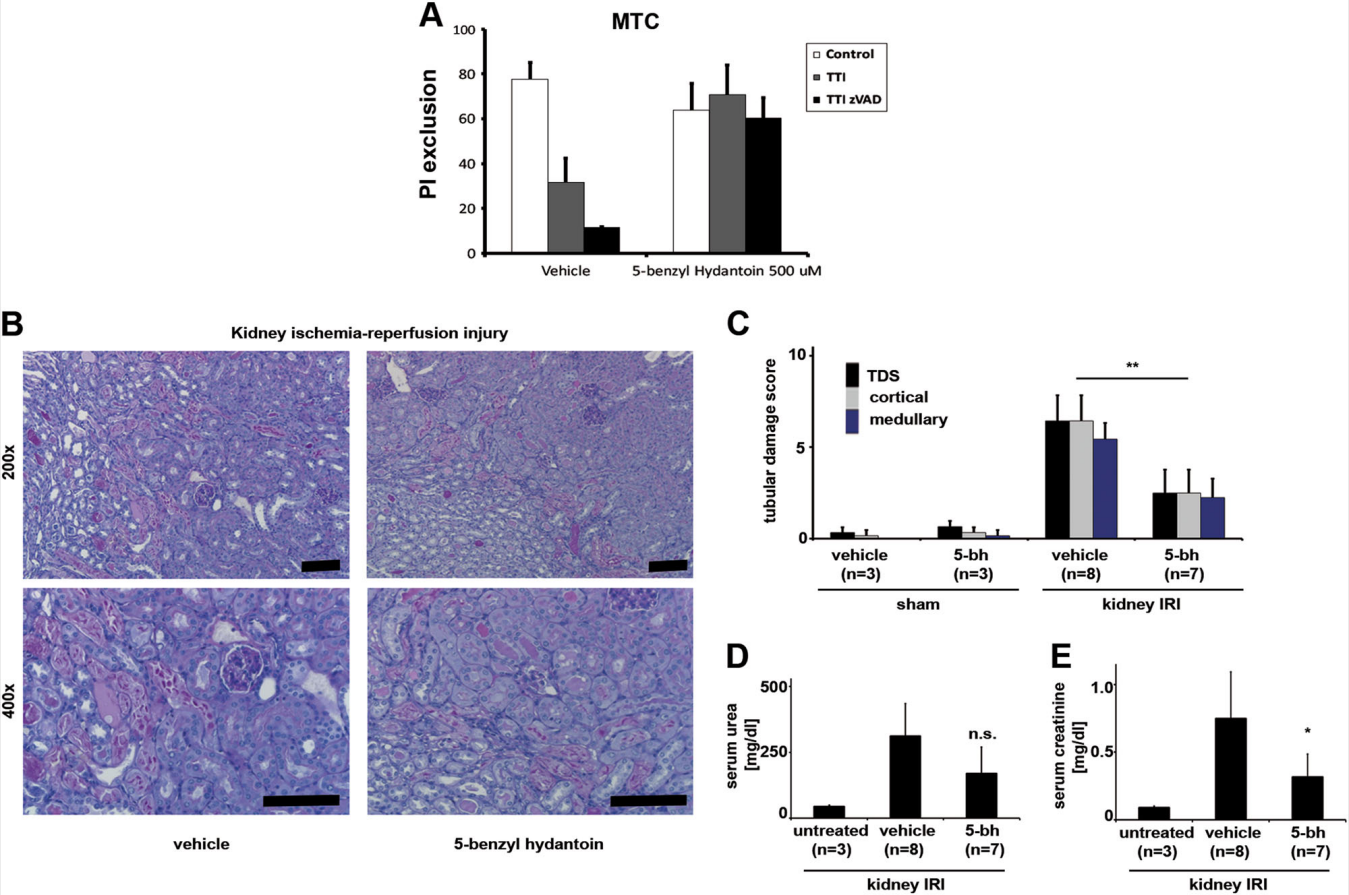
5-bezyl hydantoin (5bh) prevents necroptosis in vitro and in vivo. **a** Murine tubular cells (MCTs) were treated with TNFα/TWEAK/IFNγ plus zVAD (compare Fig. 1b) in the presence of 500 µm 5-bh and PI exclusion was measured. **b**,** c** Similar to phenytoin, and necrostatin-1 (which represents another member of the hydantoin family), 5-bh also protects against morphological criteria of renal damage in a standard model of renal IRI. In line with this finding, functional markers of acute kidney injury either demonstrated a non-significant trend towards protection (**d**) or reached statistical significance in the case of the most often used marker of serum creatinine (**e**). Statistical significance was indicated when *p* < 0.05 (*) and *p* < 0.01 (**), *n* = 7−8 per group unless otherwise indicated. Scale bars = 50 µm

As both Nec-1 and phenytoin are hydantoins, we hypothesized that other hydantoins may function similarly. We therefore investigated 5-benzyl hydantoin in the renal tubular cells treated with TTI-zVAD-fmk (compare Fig. 1b, c). Indeed, we found that also 5-benzyl hydantoin effectively prevented necroptosis in vitro (Fig. 4a) and in vivo in renal injury following kidney IRI (Fig. 4b, e).

### Phenytoin prevents necrosome formation and partially inhibits RIP1 kinase

**Fig. 5:**
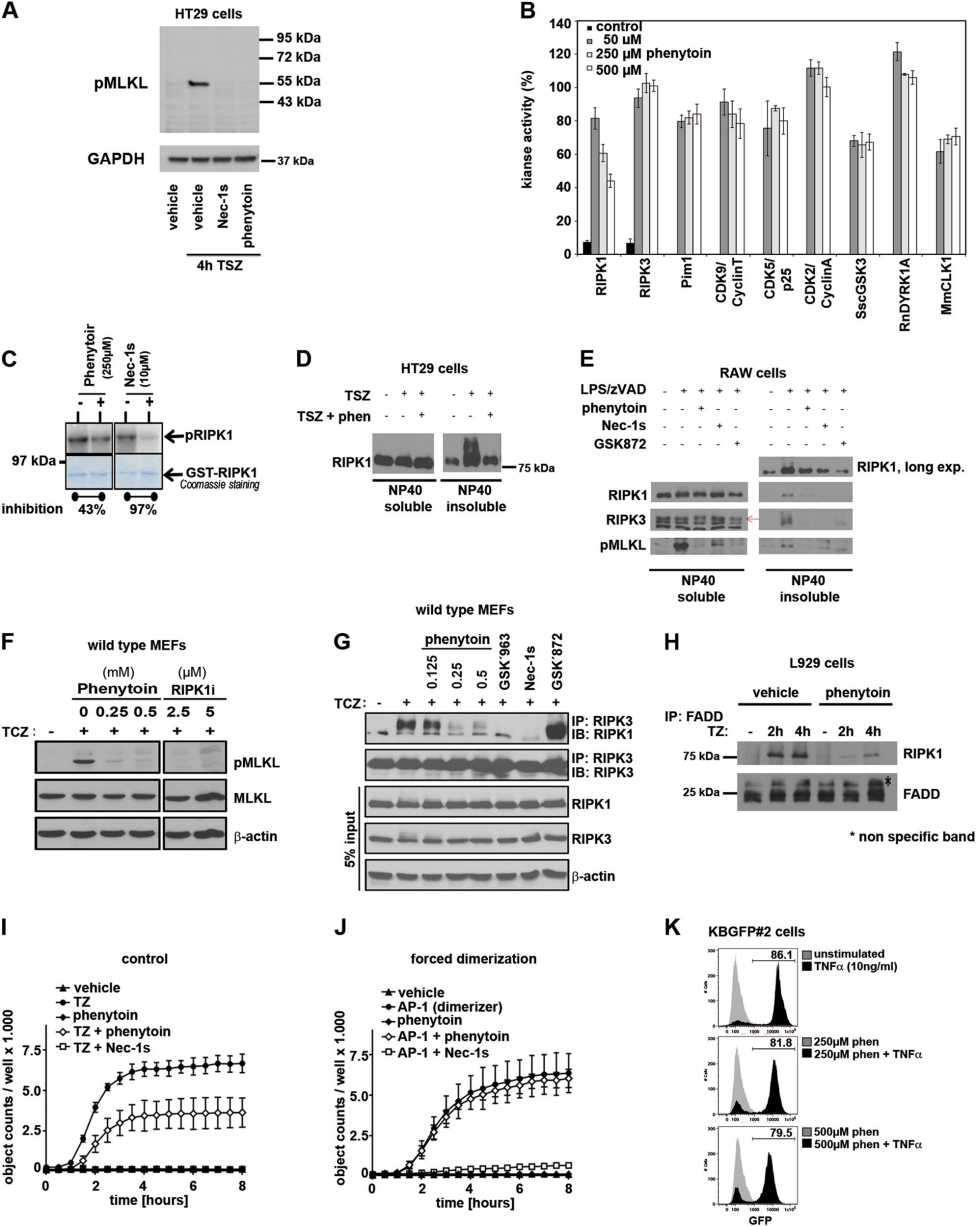
Phenytoin prevents the formation of the necrosome. **a** HT29 cells were treated with TSZ for 4 h and stained for human pMLKL. Note that phenytoin prevents the phosphorylation of MLKL as effectively as Nec-1s. **b** Screen for kinase inhibition by phenytoin reveals no major inhibition of any of the kinases investigated, including RIPK1 and RIPK3. **c** RIPK1 autophosphorylation assay as described in detail in the Methods section. 250 µm phenytoin result in 43% inhibition of RIPK1 autophosphorylation; Nec-1s serves as positive control. **d** HT-29 cells were treated with TSZ and phenytoin as indicated and NP40 soluble and insoluble fractions were separated before RIPK1 western blotting. **e** RAW cells were treated with LPS/zVAD-fmk (L/Z), phenytoin, Nec-1s and the RIPK3-inhibitor GSK872 as indicated. NP40 soluble and insoluble fractions were separated and stained for RIPK1 (short and long exposure), RIPK3 and pMLKL. **f**,** g** Primary wild-type MEFs were treated with TNF-α (50 ng/ml) in the presence of CHX (200 ng/ml) and zVAD (50 μm), with or without pre-treatment with indicated doses of phenytoin or RIPK1 inhibitor GSK’963 (indicated doses in (**f**), 5 µm in **g**) or Nec-1 (50 μm) or RIPK3 inhibitor GSK’872 (5 μm). pMLKL western blotting and RIPK3 immunoprecipitations were performed on lysates after 6 h and examined for necrosome formation by immunoblotting for RIPK1. **h** L929 cells were seeded on 100 mm plate and then stimulated with hTNF (10 ng/ml) in the presence/absence of zVAD-fmk (10 μm) and phenytoin (500 μm) for 2 and 4 h. Cells were then lysed in NP-40 lysis buffer (0.2% NP-40, 20 mm Tris, 150 mm NaCl and 10% glycerol, at pH 7.5) for 30 min in ice. Samples were centrifuged at 20,000 × *g* for 15 min and supernatants were incubated with FADD-specific antibody (M-19, Santa Cruz Biotechnology) overnight at 4 °C. Protein A/G beads (Santa Cruz Biotechnology) were added for a further 3 h. The beads were then washed five times with cold lysis buffer and FADD-associated proteins were eluted following incubating the beads in SDS-PAGE loading buffer at 70 °C for 20 min. **i**,** j** NIH3T3 + RIPK3-2xFV were incubated in absence or presence of 0.125 mm phenytoin in combination with 10 ng/ml of TNF plus 25 µm zVAD (**g**) or 2 nm AP-1 (homodimerizer; AP-20187) (**h**). Cell death was monitored by Sytox Green uptake by using an incucyte Kinetic Live Cell Imager. Alternatively, cell death was prevented by using 30 µm of Nec-1s (RIPK1 inhibitor) or 1 µm GSK’872 (RIPK3 inhibitor), respectively. **k** KBGFP#2 cells were seeded on six-well plate and then stimulated with hTNF (10 ng/ml) in the presence/absence of phenytoin (either 250 μm or 500 μm) for 24 h. Cells were then trypsinized and washed once with cold PBS. The GFP expression was analyzed by flow cytometry

Similar to Nec-1s, phenytoin prevented phosphorylation of MLKL during necroptosis, ruling out an anti-necroptotic effect on downstream mechanisms (Fig. 5a). To identify the mechanism of action of phenytoin, we investigated the effects of phenytoin on kinase activity of RIPK1 and RIPK3, by performing a kinase inhibition screen with high doses (50 µm to 500 µm) of phenytoin. The only kinase that was affected by this incubation was RIPK1 (Fig. 5b). However, in cell-free systems employing RIPK1 autophosphorylation assays (see Methods section for details), the inhibitory effect was much less pronounced when compared with the highly specific RIP1 kinase inhibitor Nec-1s (Fig. 5c). We next looked at the RIPK1 enrichment in the necrosome NP40 insoluble fractions and found that it was strongly affected by phenytoin (Fig. 5d) in HT29 cells and RAW.264 cells (Fig. 5e), as was the recruitment of RIPK3 and its oligomerization to the necrosome (Fig. 5e). We confirmed the absence of the pMLKL signal in wild-type MEFs (Fig. 5f) and employed these cells for RIPK3 immunoprecipitation to find that the formation of the necrosome was clearly reduced (Fig. 5g). In an immunoprecipitation with an anti-FADD antibody, phenytoin prevented RIPK1 recruitment to FADD in L929 cells (Fig. 5h). When we used forced RIPK3 dimerization employing the AP-1 dimerizer system, we confirmed that phenytoin prevents necrosis also in these artificially engineered cells (Fig. 5i), but forced dimerization of RIPK3 was sufficient to overcome the phenytoin-mediated protection from necroptosis (Fig. 5j). Additionally, phenytoin did not affect TNFα-mediated signaling towards the NF-κB complex (Fig. 5k).

**Fig. 6:**
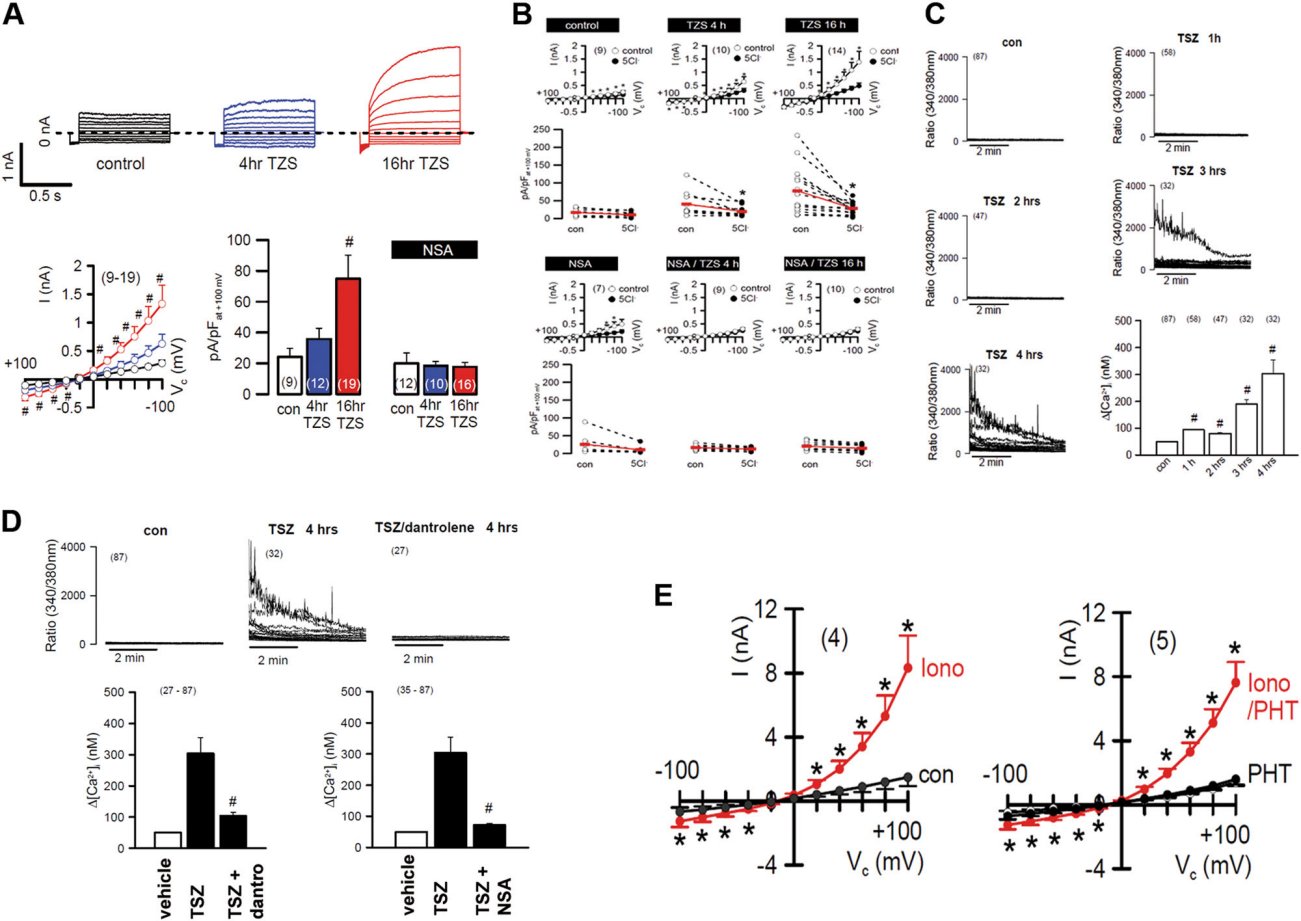
An epiphenomenal Ca^++^-current downstream of pMLKL is sensitive to dantrolene. TNFα/zVAD/SMAC (TZS)-mimetic-induced necroptosis of HT29 cells results in a whole cell current that is sensitive to necrosulfonamide (NSA) and peaks 16 h after induction (**a**). The NSA-sensitive Ca^++^-current is sensitive to chloride-depletion (**b**). TZS-induced necroptosis is accompanied by a small-scale Ca^++^-current within 3-4 h after induction of cell death (**c**) which is sensitive to dantrolene and NSA (**d**), but dantrolene does not interfere with combined positivity to annexin V and 7-AAD (compare screen in Fig. 3c). In contrast to dantrolene, however, the phenytoin prevents the cell from dying, but does not prevent the ionomycin-induced Ca^++^-signal (**e**)

We next sought to investigate the electrophysiology in the pathway of necroptosis. As expected, we detected a whole cell current during necroptosis (Fig. 6a) that was sensitive to NSA, but also to chloride (Fig. 6b). In addition, the intracellular changes in calcium concentrations during necroptosis were no higher than 300 nm (Fig. 6c) as compared to 10 µm in apoptosis. However, the Ca-signal during necroptosis was sensitive to NSA and to dantrolene (Fig. 6d), but similar concentrations of dantrolene did not affect necroptotic cell death (Fig. 3d). In contrast, phenytoin did not affect the calcium signal (Fig. 6e), but did block necroptosis (Fig. 6e). However, the dantrolene-sensitive calcium signal does not affect necroptosis and was not studied further.

In summary, these results suggest that phenytoin prevents necroptosis by an undefined mechanism that might partially involve RIP1 kinase inhibition whereas NF-kB signaling is not affected. One possibility is that RIPK1 is kept in an inactive conformation by high doses of phenytoin that may prevent the interaction with other players in the necroptosis pathway.

## Discussion

With our increasing understanding of RIPK3 biology, it becomes important to distinguish between necroptotic and non-necroptotic functions of RIPK3. Therefore, unlike RIPK3-deficiency, MLKL-deficient mice provide an ideal target to study necroptosis. We demonstrated that MLKL-dependent necroptosis contributes to the pathophysiology in mouse models of kidney IRI and SIRS, largely ruling out pathophysiologically important non-necroptotic roles of RIPK3 in these models. Given the quality of the anti-human pMLKL antibody, it is possible to detect necroptosis directly in human samples. However, even cell death independent functions of MLKL appear to contribute as MLKL-deficient mice exhibit increased peritubular blood flow.

It was previously reported that RIPK3 deficiency provides greater benefit than MLKL deficiency in mouse models of inflammation and tissue injury^16^. Our data do not support this general conclusion, at least not for kidney IRI and the TNFα-mediated SIRS, but confounding factors such as microbiota may explain these differences. However, our data presented here are in line with the conclusion that necroptosis is an important therapeutic target.

Most of the currently investigated RIPK1 inhibitors function in low micromolar range. We report that phenytoin, despite being an RIPK1 inhibitor, is at least two orders of magnitude less potent in preventing necroptosis. However, phenytoin is a hydantoin and it turns out that it displays an activity against RIPK1 kinase. This may be generally true for hydantoins as benzyl-hydantoin also displays inhibition of necroptosis. Alternatively, however, a growing body of evidence suggests that RIPK1 function are not always associated with its kinase activity, but may also be explained by kinase-independent scaffolding modes. Clearly, the necrosome assays suggest that phenytoin acts at an early step of necrosome formation, but certainly much less potent than highly specific necrostatins. Therefore, even if it was considered to perform clinical studies—because of the low costs, clinical experience, and general availability of phenytoin—data on the in vivo concentrations of phenytoin in the targeted organs are urgently awaited. Several clinical trials employing inhibitors of RIPK1 have been initiated as novel anti-necroptosis therapies emerged^3,27–30^. Despite the potential of these small molecules, none of these compounds reached phase III clinical studies today. With greater than 60 years of clinical experience and low costs alongside with the availability on ICUs and in ambulances, phenytoin may provide a drug to target necroptosis.

Finally, it remains to be investigated whether typical side effects of phenytoin that generally occur years after the onset of chronical treatment, such as gingival hyperplasia^31^, may be explained by a failure to induce necroptosis or necroinflammation^32^.

## Materials and methods

### Reagents, cell lines, and cell death assays

Necrostatin (Nec-1) was obtained from Sigma-Aldrich. The zVAD-fmk (herein referred to as zVAD) was purchased from BD Biosciences. SMAC-mimetics (Birinapant) was from Absource Diagnostics (Selleckchem). Murine NIH3T3 fibroblasts were originally obtained from ATCC and were cultured in Dulbecco’s modified Eagle medium (DMEM) (Invitrogen) supplemented with 10% (vol/vol) FCS, 100 U/ml penicillin, and 100 μg/ml streptomycin. Murine kidney cortical tubular cells (MCTs) were originally generated from the cortex of SJL mice by Eric Neilson at the University of Pennsylvania. All cell lines were cultured in a humidified 5% CO_2_ atmosphere. For induction of necroptosis, HT29 cells were stimulated for 24 h at 37 °C with 100 ng/ml TNFα plus 1 μm SMAC-mimetics plus 25 μm zVAD-fmk as indicated (vehicle-treated cells served as control). For immunoblotting, cells were lysed in ice-cold 10 mm Tris·HCl, pH 7.5, 50 mm NaCl, 1% Triton X-100, 30 mm sodium pyrophosphate, 50 mm NaF, 100 μm Na_3_VO_4_, 2 μm ZnCl_2_, and 1 mm phenylmethylsulfonyl fluoride (modified Frackelton buffer). Insoluble material was removed by centrifugation (14,000 × *g*, 10 min, 4 °C), and protein concentration was determined using a commercial Bradford assay kit according to the manufacturer’s instructions (Bio-Rad). Equal amounts of protein (17 μg per lane) were resolved on a 12% SDS/PAGE gel and transferred to a nitrocellulose membrane (Amersham Biosciences). Immune complexes were visualized by enhanced chemiluminescence (ECL; Amersham Biosciences). NIH3T3 + RIPK3-2xFV and the AP-1 dimerizer system have been described previously^19^. Cell viability was additionally assessed in RAW264.7 macrophages, seeded at 20,000 per well in 96-well format. Cells were treated for 24 h. Cell viability was assessed by total ATP release using Promega CellTiterGlo reagent. Necroptosis in MCT cells was induced by a combination of 30 ng/ml TNF, 100 ng/ml TWEAK, and 300 IU/ml Interferon-γ in the presence of 25 μm zVAD-fmk for 24 h. Primary wild-type MEFs were generated in-house from E14.5 embryos and used within ten passages in experiments. Biological and chemical reagents were from the following sources: cycloheximide (MP Biomedicals), Necrostatin 1 (BioVision), zVAD-fmk (Bachem), Phenytoin, and TNF-α (R&D Systems). Inhibitors of RIPK1 (GSK’963^33^) and RIPK3 (GSK’872^34^) from GlaxoSmithKline have been described before. Antibodies for immunoblot analysis of β-actin (Sigma), MLKL (Abgent), murine pMLKL (Abcam, ab196436), human pMLKL (Abcam, ab187091), RIPK1 (BD Transduction labs), RIPK3 (ProSci or Santa Cruz) were obtained from the indicated commercial sources.

### Fluorescence-activated cell sorting

Phosphatidylserine exposure to the outer cell membrane of apoptotic cells or at the inner plasma membrane of necrotic cells and incorporation of 7-AAD into necrotic cells were quantified by the FACS analysis. The ApoAlert annexin V-FITC antibody and the 7-AAD antibody were purchased from BD Biosciences.

### Necrosome isolation assay

Necrosome isolation assay was adapted from previously described methods^20,21^. RAW264.7 macrophages were treated for 4 h and harvested using a 1% NP40-containing lysis buffer (150 mm NaCl, 20 mm Tris-Cl (pH 7.5), 1% NP-40, 1 mm EDTA, 3 mm NaF, 1 mm B-glycerophosphate, 1 mm sodium orthovanadate, 5 μm Iodoacetamide, 2 μm*N*-ethylmaleimide) supplemented with 1 μg/ml Aproprotinin, Leupeptin, and Pepstatin, and 0.5 μg/ml PMSF. Lysates were flash frozen on dry-ice, thawed, and then centrifuged at 1000 × *g* for 10 min to precipitate nuclear matter. Supernatant was recovered and centrifuged at 34,400 × *g* to pellet NP40-insoluble matter (necrosome fraction). Resulting supernatant was collected as NP-40-soluble fraction. Necrosome fraction was boiled in 1× Laemmli buffer for 5−10 min and triturated until pellet was dispersed. NP-40-soluble and necrosome fractions were probed for RIPK1 (Cell Signaling, 3493), RIPK3 (Pro-Sci, 2283), and phospho-S345 mMLKL (Abcam, ab196436) by western analysis.

### Detection of the RIPK3 necrosome

7.5×10^5^ cells per condition were lysed in TL buffer (1% Triton X-100, 150 mm NaCl, 20 mm HEPES, 5 mm EDTA, 5 mm NaF, 0.2 mm sodium ortho-vanadate) supplemented with Complete Mini^®^ protease inhibitor cocktail (Roche) and briefly sonicated. Lysates were clarified by centrifugation at 14,000 rpm for 10 min at 4 °C, and anti-RIPK3 antibody (2 µg of ProSci or 10 µg of Santa Cruz) was added to each sample. After overnight incubation at 4 °C with rotation, samples were supplemented with 30 µl of twice-washed protein A/G-agarose slurry (Thermo Scientific) and incubated for additional 2 h, washed three times with cold TL buffer, eluted by boiling in SDS sample buffer, and resolved by SDS-PAGE. RIPK3 necrosome components were then detected by immunoblotting.

### Isolation of primary murine tubules

Six to 12 mice were used for each isolated tubule preparation, depending on the amount of material needed for particular experiments. For preparation of isolated tubules, mice were anesthetized with ketamine (100 mg/kg i.p.) and xylazine (10 mg/kg i.p.), and the kidneys were immediately removed. Type I collagenase was from Worthington Biochemical. Percoll was purchased from Amersham Biosciences. All other reagents and chemicals, including delipidated BSA, were of the highest grade available from Sigma-Aldrich. Immediately after removal of the kidneys, the parenchyma was injected with 0.3–0.5 cc of a cold 95% O_2_/5% CO_2_-gassed solution consisting of 115 mm NaCl, 2.1 mm KCI, 25 mm NaHCO_3_, 1.2 mm KH_2_PO_4_, 2.5 mm CaCl_2_, 1.2 mm MgCl_2_, 1.2 mm MgSO_4_, 25 mm mannitol, 2.5 mg/ml fatty acid-free BSA, 5 mm glucose, 4 mm sodium lactate, 1 mm alanine, and 1 mm sodium butyrate (solution A) with the addition of 1 mg/ml collagenase (type I; Northington Biochemical). The cortices were then dissected and minced on an ice-cold tile and then suspended in additional solution A for 8–10 min of digestion at 37 °C, followed by enrichment of proximal tubules using centrifugation on self-forming Percoll gradients. The fragments were transferred to collagenase solution (DS with 0.1% (wt/vol) type 2 collagenase and 96 μg/ml soybean trypsin inhibitor) and digested for 30 min at 37 °C and 850 rpm. After digestion, the supernatant was sieved through two nylon sieves: first 250-μm pore size and then 100-μm pore size. The longer proximal tubule segments remaining in the 100-μm sieve were suspended by flushing the sieve in the reverse direction with warm DS (37 °C) containing BSA 1% (wt/vol). The proximal tubule suspension was centrifuged for 5 min at 170 × *g*, washed, and then suspended into the appropriated amount of culture medium (1:1 DMEM/F12 without phenol red and supplemented with heat-inactivated 1% FCS, 15 mmol/l Hepes, 2 mmol/l l-glutamine, 50 nmol/l hydrocortisone, 5 μg/ml insulin, 5 μg/ml transferrin, 5 ng/ml sodium selenite, 0.55 mmol/l sodium pyruvate, 10 ml/l 100× nonessential amino acids, 100 IU/ml penicillin, and 100 μg/ml streptomycin buffered to pH 7.4 (osmolality of 325 mOsmol/kg H_2_O)). The proximal tubule fragments were seeded onto a tissue culture plate and cultured at 37 °C and 95% air/5% CO_2_ in a standard humidified incubator.

### Experimental procedures for, and movies of, freshly isolated tubules

Tubules were suspended at 2.0–3.0 mg of tubule protein per milliliter in a 95% air/5% CO_2_-gassed medium containing 110 mm NaCl, 2.6 mm KCl, 25 mm NaHCO_3_, 2.4 mm KH_2_PO_4_, 1.25 mm CaCl_2_, 1.2mmMgCl_2_, 1.2 mmMgSO_4_, 5 mm glucose, 4 mm sodium lactate, 0.3 mm alanine, 5 mm sodium butyrate, 2 mm glycine, and 1.0 mg/ml bovine gelatin (75 bloom) (solution B). For studies comparing normoxia with hypoxia/reoxygenation, at the end of 15 min, preincubation tubules were resuspended in fresh solution B and regassed with either 95% air/5% CO_2_ (normoxic controls) or 95% N_2_/5% CO_2_ (hypoxia). During hypoxia, solution B was kept at pH 6.9 to simulate tissue acidosis during ischemia in vivo, and the usual substrates (glucose, lactate, alanine, and butyrate) were omitted. After 30 min, samples were removed for analysis. The remaining tubules were pelleted and then resuspended in fresh 95% air/ 5% CO_2_-gassed, pH 7.4 solution B with experimental agents as needed. Sodium butyrate in solution B was replaced with 2 mm heptanoic acid during reoxygenation and supplemented with 250 μm AMP, 0.5 mg/dl delipidated albumin, and 4 mm each of α-ketoglutarate and malate. After 60 min of reoxygenation, tubules were sampled for studies. Movies with perfused isolated tubules recorded in the presence and absence of fatty acids as Q:6 indicated were taken from C57BL/6 mice, which were killed under deep isoflurane anesthesia by decapitation. Kidneys were removed immediately, sliced, and transferred into incubation buffer (140 mmol/l NaCl, 0.4 mmol/l KH_2_PO_4_, 1.6 mmol/l K_2_HPO_4_, 1 mmol/l MgSO_4_, 10 mmol/l Na-acetate, 1 mmol/l α-ketoglutarate, 1.3 mmol/l Ca-gluconate, and 5 mmol/l glycine, containing 48 mg/l trypsin inhibitor and 25 mg/l DNase I at pH 7.4). Proximal tubules (PTs) were isolated mechanically and transferred into the bath on a heated microscope stage (Axiovert 10 (PT1) or Axiovert 35 m). Tubules were held by a concentric glass pipette system. The rates of tubular perfusion via the micropipette were 10–20 nl/min. The bath was thermostated at 37 °C, and continuous bath perfusion at 3–5 ml/min was obtained by gravity perfusion. PTs were monitored by a digital imaging system (Visitron Systems GmbH) and analyzed by MetaFluor software. Brightfield images were obtained every 10–20 s and stored for off-line analysis. Solution I (140 mmol/l NaCl, 0.4 mmol/l KH_2_PO_4_, 1.6 mmol/l K_2_HPO_4_, 1 mmol/l MgCl_2_, 5 mmol/l glucose, 1.3 mmol/l Ca-gluconate, at pH 7.4) was used for fatty-acid depletion, and Solution II (140 mmol/l NaCl, 0.4 mmol/l KH_2_PO_4_, 1.6 mmol/l K_2_HPO_4_, 1 mmol/l MgSO_4_, 1.3 mmol/l Ca-gluconate, 10 mmol/l Na-acetate, 1 mmol/l α-ketoglutarate, 5 mmol/l glycine, and 5 mmol/l glucose, at pH 7.4) was used for adapted PT substrate supply.

### Ex vivo analysis of synchronized renal tubular regulated necrosis

Male MLKL-ko mice and respective male control mice were killed under deep isoflurane anesthesia by decapitation. Kidneys were removed immediately, sliced, and transferred into ice-cold dissection buffer (in mmol/l: 140 NaCl, 0.4 KH_2_PO_4_, 1.6 K_2_HPO_4_, 1 MgSO_4_, 10 Na-acetate, 1 α-ketoglutarate, 1.3 Ca-gluconate, 5 glycine, 5 glucose, containing 48 mg/l trypsin inhibitor, 25 mg/l DNase I and 1.5 g/l albumin at pH 7.4). S2 segments were isolated mechanically and transferred into the bath on a heated microscope stage (Axiovert 35 m). Tubules were held by a concentric glass pipette system^1^ and perfused with a rate of 10−20 nl/min with experimental solution (in mmol/l: 140 NaCl, 0.4 KH_2_PO_4_, 1.6 K_2_HPO_4_, 1 MgSO_4_, 1.3 Ca-gluconate, 10 Na-acetate, 1α-ketoglutarate, 5 glycine, 5 glucose, at pH 7.4) containing 50 µmol/l erastin. The bath was thermostated at 37 °C and continuous bath perfusion with experimental solution at 3−5 ml/min was obtained by gravity perfusion. PTs were monitored by a digital imaging system (Visitron Systems GmbH, Germany) and analyzed by MetaFluor software. Brightfield images were obtained every 20 s and stored for offline analysis and generation of time-lapse films.

### Mice

All WT mice reported in this study were on C57BL/6 background. Eight to 12-week-old male C57BL/6 mice (average weight ∼23 g) were used for all WT experiments, unless otherwise specified. MLKL-deficient mice were generously provided by James Murphy and Warren Alexander for the purpose of this investigation and have been described previously. All in vivo experiments were performed according to the Protection of Animals Act, after approval of the German local authorities or the Institutional Animal Care and Use Committee (IACUC) of the St. Jude Medical Department. In all experiments, mice were rigorously matched for age, sex, weight, and genetic background.

### TNFα-induced severe inflammatory response syndrome

This model has also been referred to as TNFα-induced shock by others and has been described in detail previously^4^. In our experiments, wild-type mice received intravenous (i.v.) injection of a single bolus of 25 μg murine TNFα in 200 μl PBS via the tail vein in the identical concentration in the presence or absence of phenytoin. Animals were under permanent observation and survival was checked every 30 min.

### Murine ischemia-reperfusion injury

Induction of kidney IRI was performed via a midline abdominal incision and a bilateral renal pedicle clamping for either 40 min (severe IRI, or “lethal-to-WT” IRI) or 27 min (mild IRI) using microaneurysm clamps (Aesculab). Throughout the surgical procedure, the body temperature was maintained between 36 °C and 37 °C by continuous monitoring using a temperature-controlled self-regulated heating system (Fine Science Tools). After removal of the clamps, reperfusion of the kidneys was confirmed visually. The abdomen was closed in two layers using standard 6-0 sutures. Sham-operated mice underwent the identical surgical procedures, except that microaneurysm clamps were not applied. To maintain fluid balance, all of the mice were supplemented with 1 ml of prewarmed PBS administered intraperitoneally directly after surgery. All mice were killed 48 h after reperfusion for each experiment. All ischemia-reperfusion experiments were performed in a double-blinded manner. Where indicated, phenytoin was applied intraperitoneally 15 min before the onset of ischemia in a final volume of 200 µl. In those experiments, control mice received 200 µl of PBS.

### Histology, immunohistochemistry, and evaluation of structural organ damage

Organs were dissected as indicated in each experiment and infused with 4% neutral-buffered formaldehyde, fixated for 48 h, dehydrated in a graded ethanol series and xylene, and finally embedded in paraffin. Paraffin sections (3–5 μm) were stained with periodic acid-Schiff (PAS) reagent, according to the standard routine protocol. Stained sections were analyzed using an Axio Imager microscope (Zeiss) at ×200 or ×400 magnification. Micrographs were digitalized using an AxioCam MRm Rev. 3 FireWire camera and AxioVision ver. 4.5 software (Zeiss). Organ damage was quantified by two experienced pathologists in a double-blind manner on a scale ranging from 0 (unaffected tissue) to 10 (severe organ damage). For the scoring system, tissues were stained with PAS, and the degree of morphological involvement in renal failure was determined using light microscopy. The following parameters were chosen as indicative of morphological damage to the kidney after IRI: brush border loss, red blood cell extravasation, tubule dilatation, tubule degeneration, tubule necrosis, and tubular cast formation. These parameters were evaluated on a scale of 0–10, which ranged from not present (0), mild (1–4), moderate (5 or 6), severe (7 or 8), to very severe (9 or 10). Each parameter was determined on at least four different animals.

### Intravital microscopy

In order to investigate renal hemodynamics, multiphoton microscopy experiments were performed. Mice were anesthetized by i.p. injections of ketamine/xylazine (100/10 mg/kg of body weight) before surgery was performed on an operating table with a servo-controlled heating plate. The right carotid artery was cannulated using hand-drawn polyethylene tubing so that the arterial blood pressure and the heart rate could be measured continuously. A cannula was inserted into the right jugular vein for the injection of fluorescent dyes and for an intravenous fluid supplementation over the experiment (12 µl/g body weight/h of 0.9% saline). Finally, the left kidney was exposed by making a small flank incision.

Images were then acquired using an inverted Zeiss LSM 710 NLO confocal fluorescence microscope (Carl Zeiss) equipped with a servo-controlled warming plate to maintain the body temperature of the animal at 37 °C. Excitation was achieved using a Chameleon Ultra-II MP laser (Coherent) at 860 nm.

Vasculature was labeled by injecting 80 µl of Texas Red conjugated to 70 kDa dextran (Invitrogen, 20 mg/ml stock solution purified by PD-10 Sephadex G-25M columns (GE Healthcare)) and cell nuclei were visualized using Hoechst 33342 (Invitrogen, 25 µl of a 10 mg/ml solution).

### Measurement of peritubular capillary flow

Peritubular capillary flow was investigated as described before^22,23^. Briefly, z-stacks of ten randomly chosen regions within the renal cortex were acquired per animal. Per region, the diameter and red blood cell velocity of several capillaries were measured. The capillary diameter (*d*) was determined by measuring at the widest position of the investigated vessel throughout the z-stack. By injecting 70 kDa dextran-Texas Red the plasma was labeled red, whereas the red blood cells appear as dark unstained objects inside the capillaries. The red blood cell velocity (*V*) was thereby visualized and measured by performing a time (*t*) series of 500 fast longitudinal line scans, applying a pixel dwell time of 1.97 µs. The total distance (*X*) was 20 µm within the central axis of the capillary, displaying the movement of the RBC as dark bands within the Xt-scan. The velocity (µm/ms) of the RBC is inversely proportional to the slope of the evolved bands, which was calculated as ∆*X*/∆*t*. Peritubular capillary flow (nl/min) was then calculated using the following formula:

Flow = *V* × ((*d/*2)2 × π).

### Protein kinase assays

Kinase activities were assayed in appropriate kinase buffer, with either protein or peptide as substrate in the presence of 15 µm or 30 µm (for RIPK1) [γ-^34^P] ATP (3000 Ci/mmol; 10 mCi/ml) in a final volume of 30 µl following the assay described in Bach et al.^35^. Controls were performed with appropriate dilutions of dimethylsulfoxide. Full-length kinases are used unless specified. Peptide substrates were obtained from Proteogenix (Oberhausbergen, France).

### Buffers

(A) 10 mm MgCl_2_, 1 mm EGTA, 1 mm DTT, 25 mm Tris-HCl pH 7.5, 50 µg/ml heparin; (B) 60 mm β-glycerophosphate, 30 mm p-nitrophenyl-phosphate, 25 mm MOPS (pH 7), 5 mm EGTA, 15 mm MgCl_2_, 1 mm DTT, 0.1 mm sodium orthovanadate; (R) 1.67 mm MOPS pH 7.2, 0.83 mm β-glycerophosphate, 1.33 mm MgCl_2_, 0.83 mm MnCl_2_, 0.33 mm EGTA, 0.13 mm EDTA, 16.67 μg/ml BSA, 0.017 mm DTT.

*Hs*RIPK1 autophosphorylation assay was performed following the protocol described previously^36^ by Degterev et al. Kinase reactions were assayed in buffer R with 30 µm of ATP for 30 min at 30 °C. Reactions were stopped by boiling in sample loading buffer for 3 min at 95 °C. 25 μl of each reaction was loaded per well in pre-cast NuPage 12% Bis-Tris gel (Life Technology) and analyzed by SDS-PAGE. The proteins were vizualized using Coomassie Blue staining. Typhoon PhosphorImager (GE Healthcare Life Sciences) was used to detect autophosphorylated RIPK1 band. Percent of inhibition were determined after quantification of the band intensities using Bio-Profil software (© Vilber-Lourmat).

*Hs*RIPK3 (human, recombinant, expressed by baculovirus in Sf9 insect cells) was assayed in buffer R, with 0.1 µg/µl of MBP as substrate.

*Hs*PIM1 (human proto-oncogene, recombinant, expressed in bacteria) was assayed in buffer B, with 0.8 µg/µl of histone H1 (Sigma #H5505) as substrate.

*Hs*CDK2/CyclinA (cyclin-dependent kinase-2, human, kindly provided by Dr. A. Echalier-Glazer, Leicester, UK) was assayed in buffer A (+0.15 mg/ml of BSA + 0.23 mg/ml of DTT), with 0.8 µg/µl of histone H1 as substrate.

*Hs*CDK9/CyclinT (human, recombinant, expressed by baculovirus in Sf9 insect cells) was assayed in buffer A (+0.15 mg/ml of BSA + 0.23 mg/ml of DTT), with 0.27 µg/µl of the following peptide: YSPTSPSYSPTSPSYSPTSPSKKKK, as substrate.

*Hs*CDK5/p25 (human, recombinant, expressed in bacteria) was assayed in buffer B, with 0.8 µg/µl of histone H1 as substrate.

*Ssc*GSK-3α/β (glycogen synthase kinase-3, porcine brain, native, affinity purified) was assayed in buffer A (+0.15 mg/ml of BSA + 0.23 mg/ml of DTT), with 0.010 µg/µl of GS-1 peptide, a GSK-3-selective substrate (YRRAAVPPSPSLSRHSSPHQSpEDEEE, “Sp” stands for phosphorylated serine).

*Rn*DYRK1A-kd (*Rattus norvegicus*, amino acids 1−499 including the kinase domain, recombinant, expressed in bacteria, DNA vector kindly provided by Dr. W. Becker, Aachen, Germany) was assayed in buffer A (+0.5 mg/ml of BSA + 0.23 mg/ml of DTT) with 0.033 μg/µl of the following peptide: KKISGRLSPIMTEQ as substrate.

*Mm*CLK1 (from *Mus musculus*, recombinant, expressed in bacteria) was assayed in buffer A (+0.15 mg/ml of BSA + 0.23 mg/ml of DTT) with 0.027 μg/µl of the following peptide: GRSRSRSRSRSR as substrate.

### Measurement of [Ca^++^]i and patch clamp of TSZ-stimulated cells

Measurement of the intracellular Ca2+ concentration were performed as described recently^7^. In brief, cells were loaded either with 5 μm Fura2-AM (to measure global cytosolic Ca2+ changes) in Ringer solution at 37 °C for 30 min. Fluorescence was detected at 37 °C, using an inverted microscope IMT-2 (Olympus, Nurnberg, Germany) and a high speed polychromator system (Visi- Chrome, Puchheim, Germany). The results were obtained at 340/380 nm fluorescence ratio (after background subtraction). After calibration^7^ intracellular Ca2+ concentrations were calculated.

#### Patch clamping

Cells were grown on coated glass cover slips. If not indicated otherwise, patch pipettes were filled with a cytosolic-like solution containing KCl 30, K-gluconate 95, NaH_2_PO_4_ 1.2, Na_2_HPO_4_ 4.8, EGTA 1, Ca-gluconate 0.758, MgCl_2_ 1.03, d-glucose 5, ATP 3, pH 7.2. The intracellular (pipette) Ca2+ activity was 0.1 µm. Coverslips were mounted in a perfused bath chamber on the stage of an inverted microscope (IM35, Zeiss) and kept at 37 °C. The bath was perfused continuously with Ringer solution at a rate of 8 ml/min. Patch pipettes had an input resistance of 2–4 MΩ when filled with the cytosolic-like (physiological) solution. Currents were corrected for serial resistance. The access conductance was measured continuously and was 60–140 nS. Currents (voltage clamp) and voltages (current clamp) were recorded using a patch clamp amplifier (EPC 7, List Medical Electronics, Darmstadt, Germany), the LIH1600 interface and PULSE software (HEKA, Lambrecht, Germany) as well as Chart software (AD Instruments, Spechbach, Germany). Data were stored continuously on a computer hard disc and analyzed using PULSE software. In regular intervals, membrane voltage (Vc) was clamped in steps of 20 mV from –100 to +100 mV from a holding voltage of −100 mV. Current density was calculated by dividing whole cell currents by cell capacitance.

### Human kidney biopsy samples

The human biopsies were obtained as indication biopsies for AKI at the University Hospital Cologne, Germany, strictly adhered to the statutes of the Declaration of Helsinki and the Declaration of Istanbul. Informed consent was obtained from all subjects prior to the start of the procedure.

### Statistics

For all experiments, differences of datasets were considered statistically significant when *p* values were lower than 0.05, if not otherwise specified. Statistical comparisons were performed using the two-tailed Student's *t* test. Asterisks are used in the figures to specify statistical significance (*p < 0.05; **p < 0.02; ***p < 0.001). *p* values in survival experiments (Kaplan–Meier plots) were calculated using GraphPad Prism, ver. 5.04 software. Statistics are indicated as SD unless otherwise specified.

## Electronic supplementary material

Figure S1(TIF 6858 kb)

Figure S2(TIF 920 kb)

Figure S3(TIF 1788 kb)

Figure S4(TIF 896 kb)

Supplementary Video 1(PPTX 20609 kb)

Supplementary Figure legends(DOCX 16 kb)
