# Correction to: Phenytoin inhibits necroptosis

**DOI:** 10.1038/s41419-018-0638-2

**Published:** 2018-05-24

**Authors:** Anne von Mässenhausen, Wulf Tonnus, Nina Himmerkus, Simon Parmentier, Danish Saleh, Diego Rodriguez, Jiraporn Ousingsawat, Rosalind L. Ang, Joel M. Weinberg, Ana B. Sanz, Alberto Ortiz, Adrian Zierleyn, Jan Ulrich Becker, Blandine Baratte, Nathalie Desban, Stéphane Bach, Ina Maria Schiessl, Shoko Nogusa, Siddharth Balachandran, Hans Joachim Anders, Adrian T. Ting, Markus Bleich, Alexei Degterev, Karl Kunzelmann, Stefan R. Bornstein, Douglas R. Green, Christian Hugo, Andreas Linkermann

**Affiliations:** 1 Division of Nephrology, Medical Clinic 3, University Hospital Carl Gustav Carus, Technical University Dresden, Dresden, Germany; 2 Department of Physiology, University of Kiel, Kiel, Germany; 3 Sackler School of Graduate Biomedical Sciences, Tufts University School of Medicine, Boston, MA USA; 4 Department of Immunology, St. Jude Medical Research Hospital, Memphis, TN USA; 5 Institute for Physiology, University of Regensburg, Regensburg, Germany; 6 Immunology Institute, Icahn School of Medicine at Mount Sinai, New York, NY 10029 USA; 7 Department of Internal Medicine, University of Michigan, Ann Arbor, MI United States; 8 Nephrology, IIS-Fundacion Jimenez Diaz UAM, FRIAT and REDINREN, Madrid, Spain; 9 Institute of Pathology, University of Cologne, Cologne, Germany; 10 Protein Phosphorylation and Human Disease Laboratory, Station Biologique de Roscoff UPMC Univ Paris 06 CNRS USR3151, CS 90074, Sorbonne Universités, 29688 Roscoff Cedex, France; 11 Blood Cell Development and Function Program, Fox Chase Cancer Center, Philadelphia, PA USA; 12 Medizinische Klinik und Poliklinik IV, Klinikum der LMU München, Munich, Germany

**Correction to**: *Cell Death and Disease* (2018) **9**:359. 10.1038/s41419-018-0394-3; Article published online: 02 Mar 2018.

The name of the one of the authors was misspelt. The author’s surname is Rodriguez, not Rodriquez as originally published. This has been corrected in both the PDF and HTML versions of the Article.

