# Supplementary figures and images for: Phenytoin inhibits necroptosis
Source: Cell Death Dis. 2018 Mar 2;9(3):359. doi: 10.1038/s41419-018-0394-3 (PMC5834524; doi:10.1038/s41419-018-0394-3)

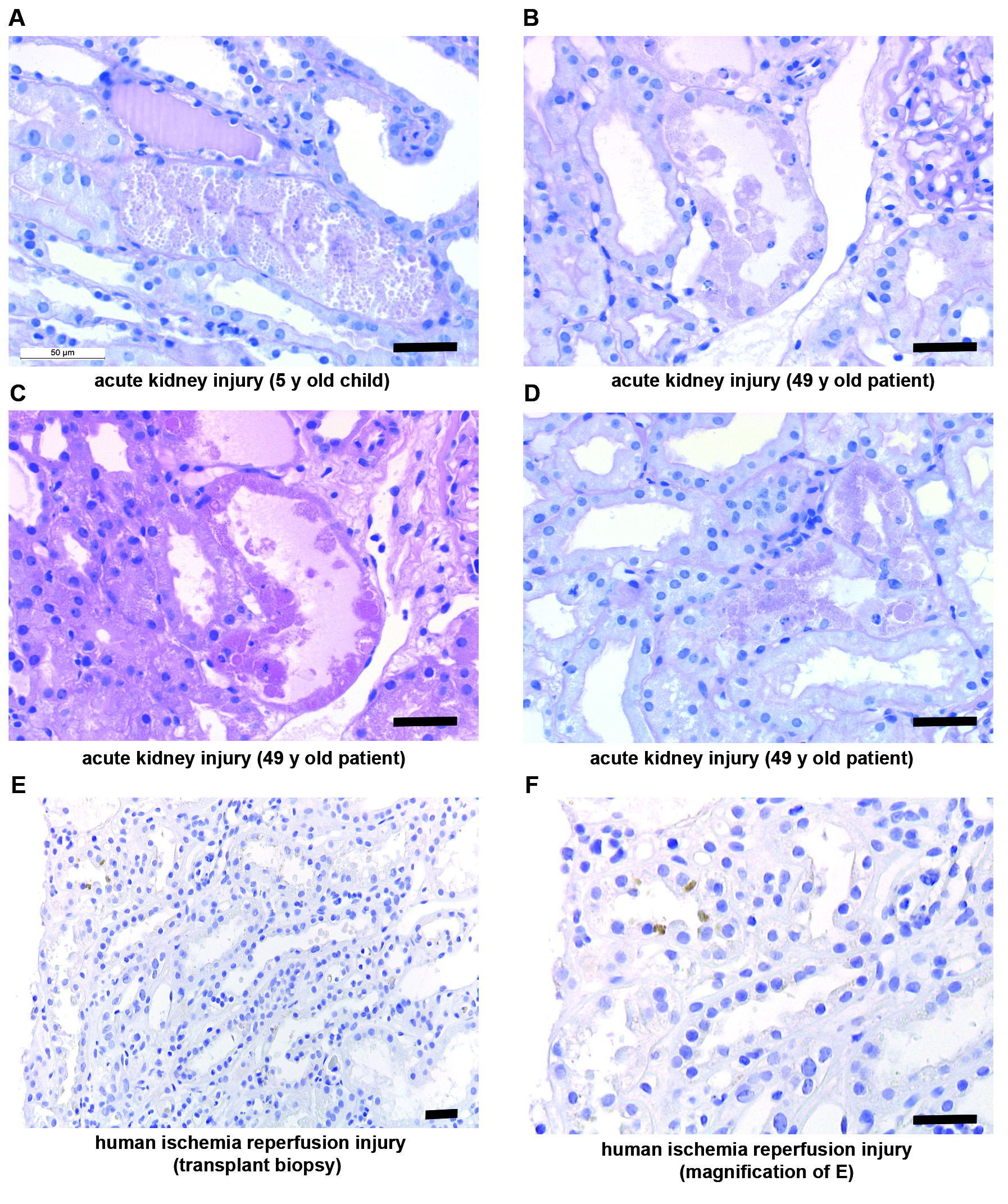

Supplement: Supplementary file 1 — Figure S1(TIF 6858 kb) [file 41419_2018_394_MOESM1_ESM.tif]

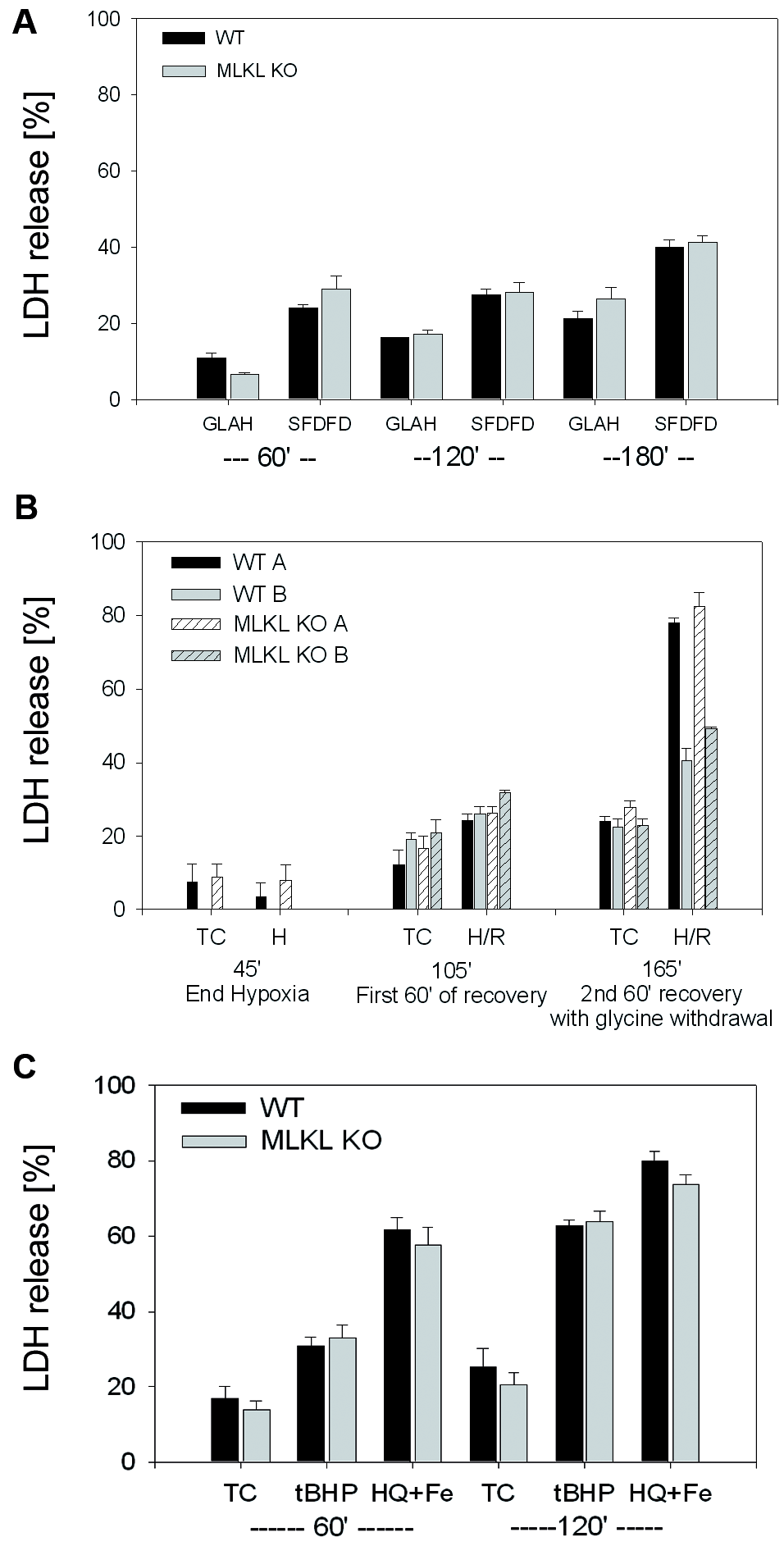

Supplement: Supplementary file 2 — Figure S2(TIF 920 kb) [file 41419_2018_394_MOESM2_ESM.tif]

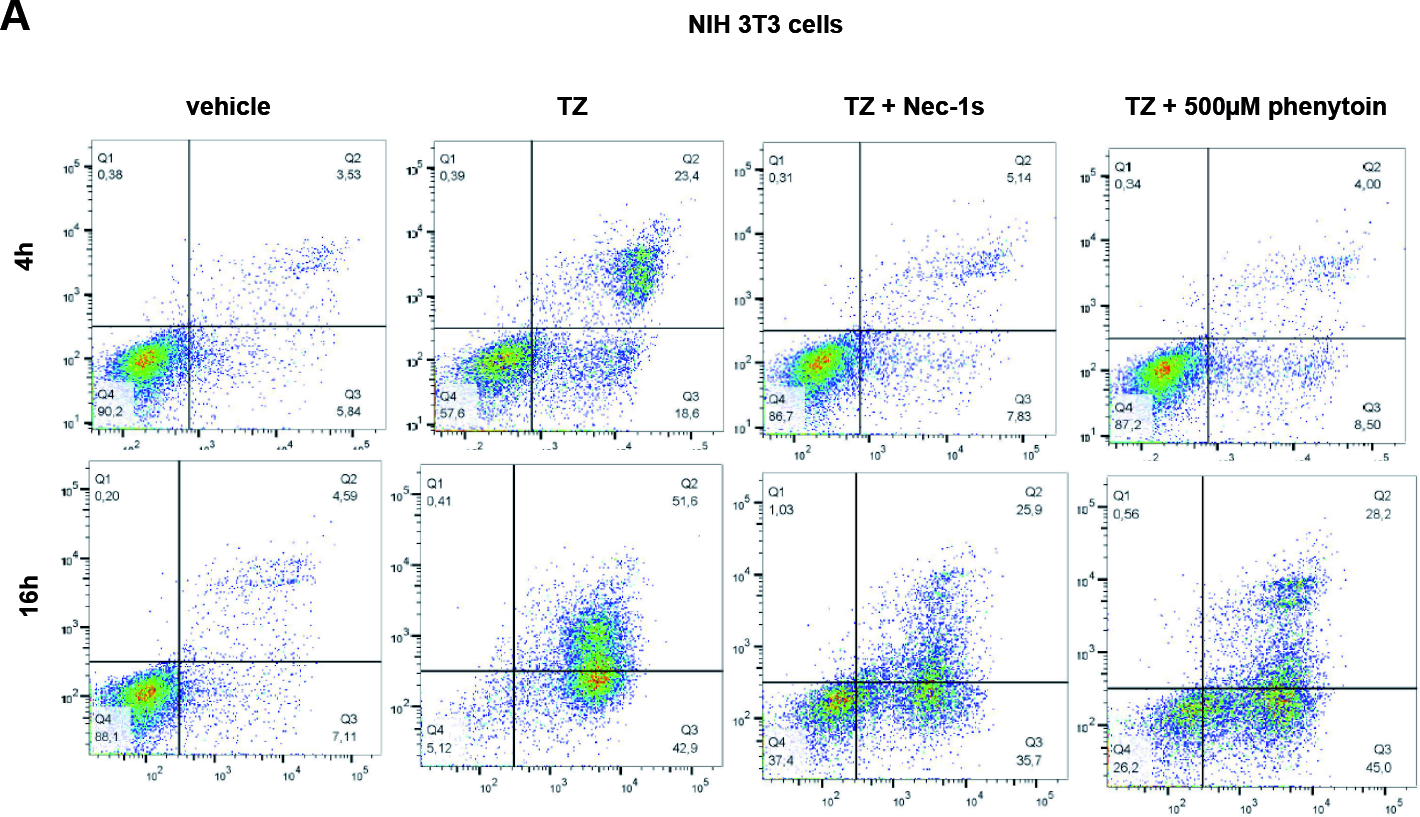

Supplement: Supplementary file 3 — Figure S3(TIF 1788 kb) [file 41419_2018_394_MOESM3_ESM.tif]

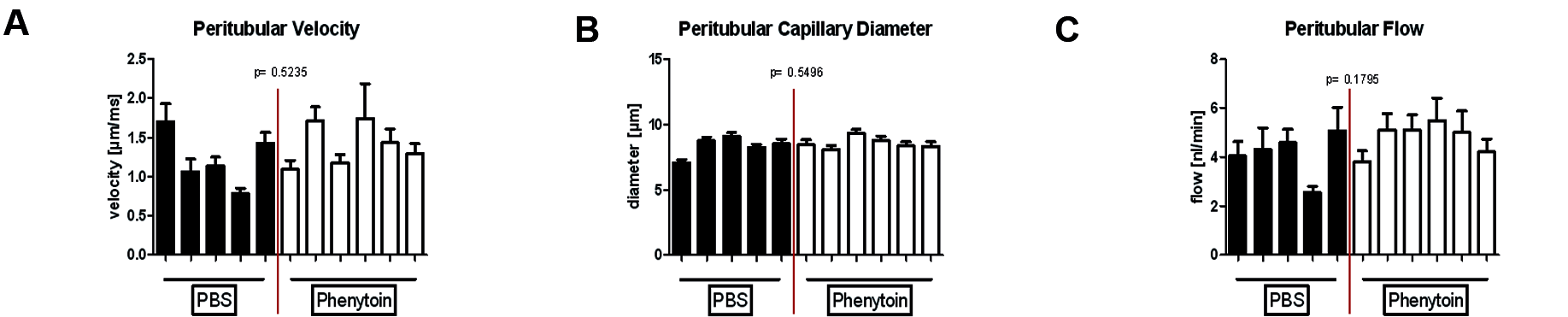

Supplement: Supplementary file 4 — Figure S4(TIF 896 kb) [file 41419_2018_394_MOESM4_ESM.tif]

## Slide 1
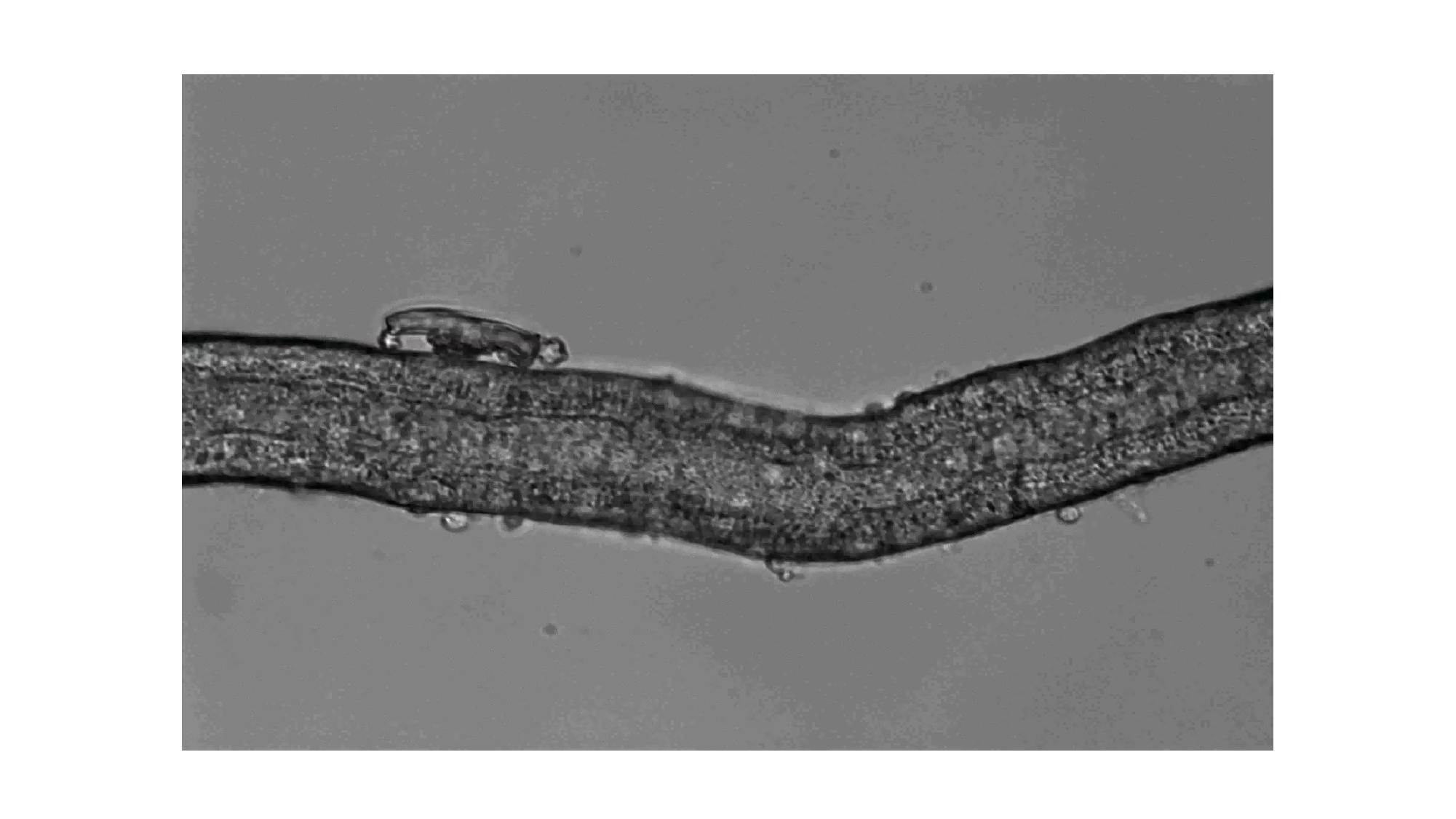

Supplement: Supplementary file 5 — Supplementary Video 1(PPTX 20609 kb) [file 41419_2018_394_MOESM5_ESM.pptx]
