# Supplementary material for: Phenytoin inhibits necroptosis
Source: Cell Death Dis. 2018 Mar 2;9(3):359. doi: 10.1038/s41419-018-0394-3 (PMC5834524; doi:10.1038/s41419-018-0394-3)
Supplement: Supplementary file 6 — Supplementary Figure legends(DOCX 16 kb) [file 41419_2018_394_MOESM6_ESM.docx]

**Supplementary Information to**

**Phenytoin inhibits receptor interacting protein kinase 1 and prevents necroptosis *in vivo*.**

**Supplementary Legends and Figures**

**Figure S1: Necrosis, such as synchronized tubular necrosis (STN), represents a typical feature of acute kidney injury.** Periodic acid Schiff (PAS)-staining of a kidney biopsy obtained from a 5 year old child suffering from acute kidney injury reveals STN (**S1A**). PAS-staining of a kidney biopsy taken from a 49 year old patient who suffered from acute kidney injury following intoxication with anesthetics shows multiple loci of STN (**S1B-S1D**). Immunohistochemistry for human pMLKL of a renal allograft 4 days following transplantation reveals a very clear background of the antibody (**S1E**) and clearly demonstrates positivity in few tubules that are about to undergo regulated necrosis (**S1F**). Scale bars = 50 µm

**Figure S2** (related to Figure 2)**: Studies of kidney proximal tubules from wild type and MLKL knockout (KO) mice subjected to 3 different insults.** Tubules were incubated for 180 minutes with either the usual full substrate mixture (glucose, lactate, alanine and hepatanoate – GLAH) or in substrate-free (SF) medium with sampling for LDH every 60 min. Substrate-free medium increased the background injury that occurred spontaneously over this period. Tubules from MLKL KO mice did not differ from WT. Values are means±SEM for n=4 (**A**). In (**B**), tubules were subjected to either time control incubation (TC) or to 15 minutes preincubation then 30 minutes hypoxia (H) followed by 120 min. reoxygenation (R). LDH release was measured at the end of the hypoxic period (45’ total incubation), then after 60 min. reoxygenation (105’ total incubation), and after 120 min. reoxygenation (165’ total incubation). The reoxygenation medium had either only the usual full substrate mixture (condition ‘A’) or was further supplemented with 4 mM a-ketoglutarate+malate + 0.5 mg/ml delipidated albumin (condition ‘B’) to promote mitochondrial recovery. The cytoprotective amino acid, glycine (2 mM), was present until the end of the first 60 min. reoxygenation, then was withdrawn for the final 60 min. to unmask cell killing in tubules that had not metabolically recovered. This is seen in ‘A’ flasks when glycine is withdrawn during the second 60 min. of reoxygenation. Tubules from MLKL KO mice did not differ from WT during any phase of the experiment. Values are means±SEM for n=4. In (**C**), ferroptosis was induced by exposure of tubules to either 5 mM tert-butylhydroperoxide (tBHP) or 10 mM each of hydroxyquinoline and ferrous ammonium sulfate. TC (time control without any experimental additions). Samples were assayed for LDH release after 60 and 120 min. exposure. Tubules from MLKL KO mice did not differ from WT. Values are means±SEM for n=4.

**Figure S3** (related to Figure 3)**:** (**A**) Verification of phenytoin in NIH3T3 cells as an inhibitor of TZ-induced necroptosis in direct comparison with Nec-1.

**Figure S4** (related to Figure 3)**: Phenytoin does not affect peritubular flow.** Peritubular velocity (**A**), peritubular capillary diameter (**B**) and peritubular flow (**C**) were determined by intravital microscopy as explained in the methods section. No statistically significant differences were documented in each of these parameters upon addition of 250µM phenytoin (n=5 mice in PBS group vs. 6 mice in phenytoin group).

**Supplementary video legend**

**Video S1: Morphological changes upon erastin-infusion.** Freshly-isolated hand-picked renal tubules obtained from wild type mice were perfused with erastin. Morphological changes are visible in the video. Videos were used to quantify the occurrence rate of ballooning cells and the onset of synchronized tubular necrosis (STN).
